# Genome-Wide Transcriptional Regulation and Chromosome Structural Arrangement by GalR in *E. coli*

**DOI:** 10.3389/fmolb.2016.00074

**Published:** 2016-11-16

**Authors:** Zhong Qian, Andrei Trostel, Dale E. A. Lewis, Sang Jun Lee, Ximiao He, Anne M. Stringer, Joseph T. Wade, Thomas D. Schneider, Tim Durfee, Sankar Adhya

**Affiliations:** ^1^Laboratory of Molecular Biology, National Institutes of Health, National Cancer InstituteBethesda, MD, USA; ^2^Microbiomics and Immunity Research Center, Korea Research Institute of Bioscience and BiotechnologyDaejeon, Korea; ^3^Laboratory of Metabolism, National Institutes of Health, National Cancer InstituteBethesda, MD, USA; ^4^Wadsworth Center, New York State Department of HealthAlbany, NY, USA; ^5^Department of Biomedical Sciences, School of Public Health, University of AlbanyAlbany, NY, USA; ^6^Gene Regulation and Chromosome Biology Laboratory, National Institutes of Health, National Cancer Institute, Center for Cancer ResearchFrederick, MD, USA; ^7^DNASTAR, Inc.Madison, WI, USA

**Keywords:** GalR regulon, mega-loop, ChIP-chip, nucleoid, DNA superhelicity

## Abstract

The regulatory protein, GalR, is known for controlling transcription of genes related to D-galactose metabolism in *Escherichia coli*. Here, using a combination of experimental and bioinformatic approaches, we identify novel GalR binding sites upstream of several genes whose function is not directly related to D-galactose metabolism. Moreover, we do not observe regulation of these genes by GalR under standard growth conditions. Thus, our data indicate a broader regulatory role for GalR, and suggest that regulation by GalR is modulated by other factors. Surprisingly, we detect regulation of 158 transcripts by GalR, with few regulated genes being associated with a nearby GalR binding site. Based on our earlier observation of long-range interactions between distally bound GalR dimers, we propose that GalR indirectly regulates the transcription of many genes by inducing large-scale restructuring of the chromosome.

## Introduction

The 4.6 Mb *Escherichia coli* chromosomal DNA is packaged into a small volume (0.2–0.5 μm^3^) for residing inside a cell volume of 0.5–5 μm^3^ (Loferer-Krossbacher et al., 1998; Skoko et al., 2006; Luijsterburg et al., 2008). It has been suggested that a bacterial chromosome has a 3-D structure that dictates the entire chromosome's gene expression pattern (Kar et al., 2005; Macvanin and Adhya, 2012). The chromosome structure and the associated volume are defined and environment-dependent. The compaction of the DNA into a structured chromosome (nucleoid) is facilitated by several architectural proteins, often called “nucleoid-associated proteins” (NAPs). NAPs are well-characterized bacterial histone-like proteins such as HU, H-NS, Fis, and Dps (Ishihama, 2009). For example, deletion of the gene encoding the NAP HU leads to substantial changes in cell volume and in the global transcription profile, presumably due to changes in chromosome architecture (Kar et al., 2005; Oberto et al., 2009; Priyadarshini et al., 2013). A recent and surprising addition to the list of NAPs in *E. coli* is the sequence-specific DNA-binding transcription regulatory protein, GalR (Qian et al., 2012). In contrast, related DNA-binding proteins PurR, MalT, FruR, and TyrR do not appear to affect the chromosome structure (Qian et al., 2012). Here, we discuss experimental results that led us to explore the idea that GalR also regulates transcription at a global scale through DNA architectural changes.

**Figure 1:**
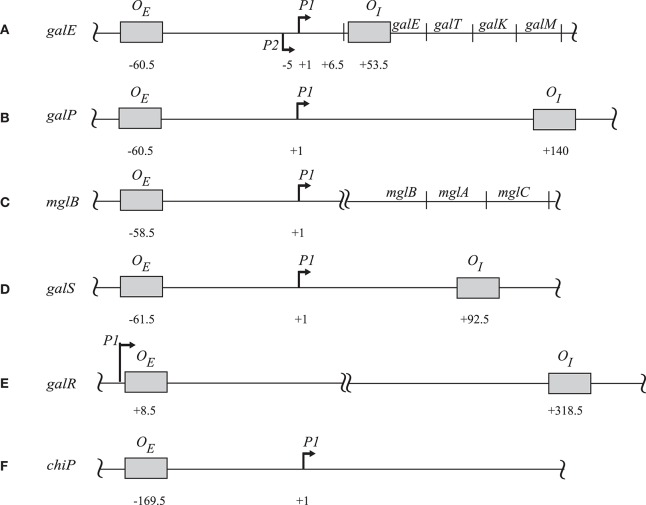
**The regulatory sites of (A)** *galE*, **(B)** *galP*, **(C)** *mglB*, **(D)** *galS*, **(E)** *galR*, and **(F)** *chiP* operons. The operator sites in each operon are in shaded boxes with their locations relative to the corresponding *tsp*. For *galE* the *tsps* are indicated as (+1) for *P1* and (−5) for *P2*. The amino terminus of the first protein in each operon is indicated by a double-arrow. The diagram is not drawn to scale (Hogg et al., 1991; Weickert and Adhya, 1993a,b; Plumbridge et al., 2014).

**Figure 2:**
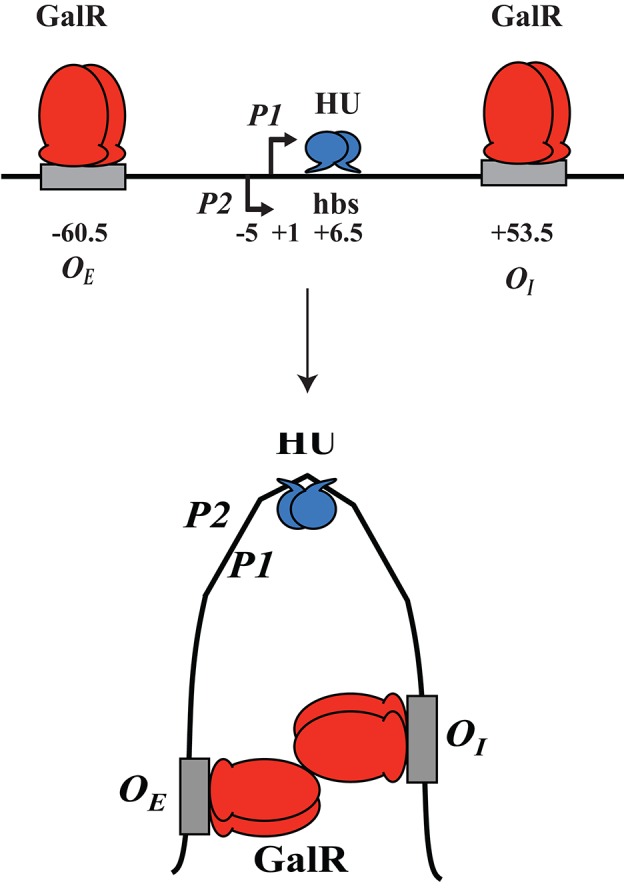
**DNA looping. GalR (red) binds to both ***O***_***E***_and ***O***_***I***_; ***hbs*** reflects the HU binding site at position +6.5**. GalR-*O*_*E*_and GalR-*O*_*I*_ interact, the intervening DNA loops out and forms a kink, while HU (blue) binds and stabilizes the loop.

GalR regulates transcription of the *galETKM, galP, galR, galS*, and *mglBAC* transcripts (Figure 1). These genes all encode proteins involved in the transport and metabolism of D-galactose. Moreover, GalR controls expression of the *chiPQ* operon, which encodes genes involved in the transport of chitosugar. The *galETKM* operon (Figure 1) is transcribed as a polycistronic mRNA from two overlapping promoters, *P1* (+1) and *P2* (−5) (Musso et al., 1977; Aiba et al., 1981). GalR regulates *P1* and *P2* promoters differentially. GalR binds two operators, *O*_*E*_, located at position −60.5, and *O*_*I*_, located at +53.5 (Irani et al., 1983; Majumdar and Adhya, 1984, 1987). Binding of GalR to *O*_*E*_ represses *P1* and activates *P2* by arresting RNA polymerase, and facilitating the step of RNA polymerase isomerization, respectively (Roy et al., 2004). When GalR binds to both *O*_*E*_ and *O*_*I*_, which are 113 bp apart and do not overlap with the two promoters, it prevents transcription initiation from both *P1* and *P2* (Aki et al., 1996; Aki and Adhya, 1997; Semsey et al., 2002; Roy et al., 2005). Mechanistically, two DNA-bound GalR dimers transiently associate, creating a loop in the intervening promoter DNA segment. Kinking at the apex of the loop facilitates binding of HU, which in turn stabilizes the loop (Figure 2; Kar and Adhya, 2001). The DNA structure in the looped form is topologically closed and binds RNA polymerase, but does not allow isomerization into an actively transcribing complex (Choy et al., 1995).

**Figure 3:**
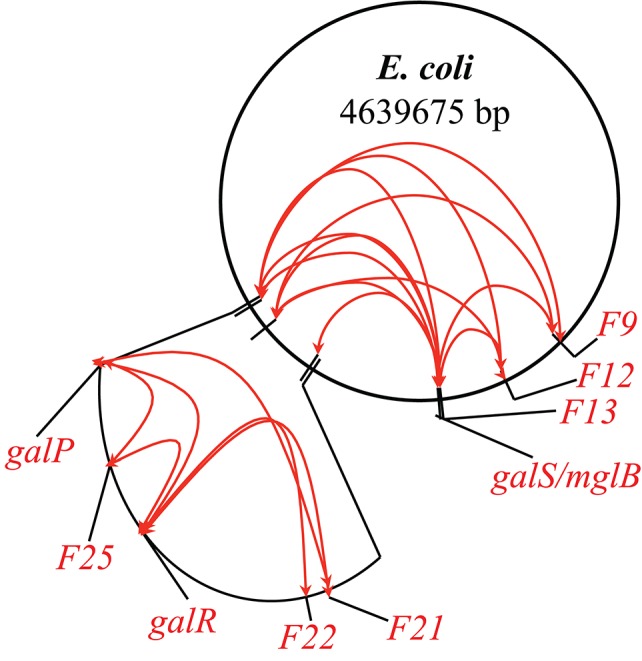
**Inter-segmental DNA networks by GalR in ***E. coli*****. The network was determined by 3C assays (see text) and is shown by red lines. Only a subset of the GalR-mediated intersegmental operator connections are shown.

Following the example of GalR-mediated DNA loop formation by interaction of GalR bound to two operators in the *galE* operon, and considering the fact that GalR operators in the *galP, mglB, galS, galR*, and *chiP* promoters are scattered around the chromosome, we hypothesized that GalR may oligomerize while bound to distal sites, thereby forming much larger DNA loops (“mega-loops”). We employed the Chromosome Conformation Capture (3C) method to investigate interactions between distal GalR operators (Dekker et al., 2002). Thus, we showed that GalR does indeed oligomerize over long distances, resulting in the formation of mega-loops. Moreover, our data suggested the existence of other unidentified GalR binding sites around the chromosome, with these novel sites also participating in long-distance interactions (Qian et al., 2012). Figure 3 shows in a cartoon from the demonstrable GalR-mediated DNA-DNA connections as listed in Table 1. Although, we originally proposed that DNA-bound GalR-mediated mega-loops may serve to increase the local concentrations of GalR around their binding sites for regulation of the adjacent promoters (Oehler and Muller-Hill, 2010), global regulation of gene expression due to change in chromosome structure may be another consequence of mega-loop formation. We propose that GalR-mediated mega-loop formation results in the formation of topologically independent DNA domains, with the level of superhelicity in each domain influencing transcription of the local promoters.

**Table 1:** **List of GalR operators identified by 3C method**.

**Chromosome**	**Coordinates**	**Operators**	**DNA sequence(s)**
3088004	3088019	*O_*E*_* (*galP*)	CTGAAACCGATTACAC
3088186	3088201	*O_*I*_* (*galP*)	GTGTAATCGCTTACAC
2976569	2976584	*O_*E*_* (*galR*)	ATGTAAGCGTTTACCC
2976830	2976845	*O_*I*_* (*galR*)	GTTCGACCGCTTTCAC
2240618	2240633	*O_*E*_* (*galS*)	TTGAAAGCGGTTACAT
2241611	2241626	*O_*I*_* (*galS*)	GGGAAACCGTTGCCAC
2239532	2239547	*O* (*mglB*)	GTGCACCGGATTTCAC
1737872	1737887	*O* (*F9*)	GTGGAAACGTTTGCTC
1990112	1990127	*O* (*F12*)	ATTTAACCGTTTTCTG
2246944	2246959	*O* (*F13*)	TTGTTATCGTTTGCAT
2738456	2738471	*O* (*F21*)	ATGGAAAAGGTTGCAC
2783816	2783831	*O* (*F22-1*)	GCGAAAACGGTTTAAG
2784177	2784192	*O* (*F22-2*)	CTGCAAGCTTTTTCCA
2786317	2786332	*O* (*F22-3*)	TTGCAATTACTTTCAC
3072949	3072964	*O* (*F25-1*)	CTTAAATCGATTGCCG
3072989	3073004	*O* (*F25-2*)	TTTGAAGCGATTGCGG
3073430	3073445	*O* (*F25-3*)	CTGCAATCGCTCCCCT

*Connections were detected among these sites except galE_E_ and galE_I_ by 3C assays. The first seven operators that showed connections by 3C were known before. The ones named as F were discovered during the 3C studies (Qian et al., 2012)*.

## Materials and methods

### Bacterial and bacteriophage strains

Bacteriophage P1 lysates of *galR::kanR* (from Keio collection; (Baba et al., 2006)) were made and *E. coli* K-12 MG1655 *galR* deletion strains were constructed from MG655 by bacteriophage P1 transduction using the lysate. Cells were then grown in 125 ml corning flasks (Corning® 430421) containing 30 ml of M63 minimal medium plus D-fructose (final concentration 0.3%) at 37°C with 230 rpm shaking. At OD600 0.6, cell cultures were separated into two flasks. Subsequently, D-galactose (final concentration 0.3%) or water was added and cells were cultivated for an additional 1.5 h at 37°C.

*E. coli* MG1655 *galR*-TAP (AMD032) was constructed by bacteriophage P1 transduction of the *kanR*-linked TAP tag cassette from DY330 *galR*-TAP (Butland et al., 2005). The *kanR* cassette was removed using pCP20, as described previously (Datsenko and Wanner, 2000). *E. coli* MG1655 *galR*-FLAG_3_ (AMD188) was constructed using FRUIT (Stringer et al., 2012).

### RNA isolation

Cell cultures were placed on ice and RNAprotect™ Bacteria Reagent (Qiagen® 76506) was added to stabilize the RNA (Lee et al., 2014). Cells were harvested for RNA purification by RNeasy® Mini Kit (Qiagen® 74104) following the manufacturer's recommendations. RNA concentrations and purity were measured using a Thermo Scientific NanoDrop™ 1000. Further sample processing was performed according to the Affymetrix GeneChip® Expression Analysis Technical Manual, Section 3: Prokaryotic Sample and Array Processing (701029 Rev.4).

### cDNA synthesis

Isolated RNA (10 μg) was used for Random Primer cDNA synthesis using SuperScript II™ Reverse Transcriptase (Invitrogen Life Technologies 18064-071). The reaction mixture was treated with 1N NaOH to degrade any remaining RNA and treated with 1N HCl to neutralize the NaOH. Synthesized cDNA was then purified using MinElute® PCR Purification columns (Qiagen® 28004). Purified cDNA concentration and purity were measured using a Thermo Scientific NanoDrop™ 1000.

### cDNA fragmentation

Purified cDNA was fragmented to between 50 and 200 bp by 0.6 U/μg of DNase I (Amersham Biosciences 27-0514-01) for 10 min at 37°C in 1X One-Phor-All buffer (Amersham Biosciences 27-0901-02). Heat inactivation of the DNase I enzyme was performed at 98°C for 10 min.

### cDNA labeling

Fragmented cDNA was then 3′ termini biotin labeled using the GeneChip® DNA Labeling Reagent (Affymetrix 900542) and 60 U of Terminal Deoxynucleotidyl Transferase (Promega M1875) at 37°C for 60 min. The labeling reaction was then stopped by the addition of 0.5 M EDTA.

### Microarray hybridization

Labeled cDNA fragments (3 μg) were then hybridized for 16 h (60 rpms) at 45°C to tiling array chips (Ecoli_Tab520346F) purchased from Affymetrix (Santa Clara, CA). The chips have 1,159,908 probes in 1.4 cm × 1.4 cm and a 25-mer probe every 8 bps in both strands of whole *E. coli* genome. In addition, the probes are also overlapped by 4 bps with other strand probes. Each 25-mer DNA probe in the tiling array chip are 8 bp apart from the next probe. Probes are designed to cover the whole *E. coli* genome.

### Microarray: washing and staining

The chips were then washed with Wash Buffer A: Non-Stringent Wash Buffer (6X SSPE, 0.01% Tween-20). Wash Buffer B: (100 mM MES, 0.1M [Na^+^] and 0.01%Tween-20) and stained with Streptavidin Phycoerythrin (Molecular Probes S-866) and anti-streptavidin antibody (goat), biotinylated (Vector Laboratories BA-0500) on a Genechip Fluidics Station 450 (Affymetrix) according to washing and staining protocol, ProkGE-WS2_450.

### Microarray: scanning and data analysis

Hybridized, washed, and stained microarrays were scanned using a Genechip Scanner 3000 (Affymetrix). Standardized signals, for each probe in the arrays, were generated using the MAT analysis software, which provides a model-based, sequence-specific, background correction for each sample (Johnson et al., 2006). A gene specific score was then calculated for each gene by averaging all MAT scores (natural log) for all probes under the annotated gene coordinates. Gene annotation was from the ASAP database at the University of Wisconsin-Madison, for *E. coli* K-12 MG1655 version m56 (Glasner et al., 2003). Data were graphed with ArrayStar®, version 2.1. DNASTAR. Madison, WI. The tiling array data was submitted to NCBI Gene Expression Omnibus. The accession number is GSE85334.

### ChIP-chip assays

MG1655 *galR*-TAP (AMD032) cells were grown in LB at 37°C to an OD_600_ of ~0.6. ChIP-chip was performed as described previously (Stringer et al., 2014). Data analysis was performed as described previously except that probes were ignored only if they had a score of <100 pixels, indicating regions that are likely missing from the genome (Stringer et al., 2014). Adjacent probes scoring above the threshold for being called as being in GalR-bound regions were merged, and the highest-scoring probe was selected as the “peak position.” The closely spaced peaks upstream of *mglB* and *galS* were manually separated. The ChIP-chip data was submitted to the EBI Array Express repository. The accession number is E-MTAB-4903.

### Identification of an enriched sequence motif from ChIP-seq data

For each peak position, we extracted genomic DNA sequence using the following formulae to determine the upstream and downstream coordinates: upstream coordinate: U_*P*_−((U_*P*_−U_*P*−1_) ^*^ (S_*P*−1_ / S_*P*_)); downstream coordinate: D_*P*_−((D_*P*+1_−D_*P*_) ^*^ (S_*P*+1_/S_*P*_)); where S = probe score, U = genome coordinate corresponding to the upstream end of a probe, D = genome coordinate corresponding to the downstream end of a probe, _*P*_ = peak probe, _*P*−1_ = probe upstream of peak, and _*P*+1_ = probe downstream of peak. We used MEME (version 4.11.2, default parameters except any number of motif repetitions was allowed) to identify an enriched sequence motif (Bailey and Elkan, 1994).

### ChIP-qPCR

MG1655 *galR*-FLAG_3_ (AMD188) cells were grown in LB at 37°C to an OD_600_ of 0.6–0.8. ChIP-qPCR was performed as described previously (Stringer et al., 2014).

## Results

### *In silico* identification of novel GalR target genes in *E. coli*

A consensus sequence of GalR binding sites from the previously known functional 9 operators in the *gal* regulon (*galE, galP, mglB, galS*, and *galR* promoters; Figure 1) appears to be a 16-bp hyphenated dyad symmetry sequence with the center between positions 8 and 9: ^1^GT**G**N**A**ANC.**G**NTTNC**A**C^16^ (with N being any nucleotide; Weickert and Adhya, 1993a). Genetic analysis showed that mutations at any of the positions 3, 5, 9, and 15 (labeled in bold) create a functionally defective operator (Adhya and Miller, 1979). Therefore, we used a motif in which nucleotides at positions 3, 5, 9, and 15 were fixed to search through the whole genome of *E. coli* (NC_000193.3) (Baba et al., 2006) for putative GalR operators, allowing two mismatches at other non-N positions as described (Qian et al., 2012). Thus, we found 165 potential GalR operators distributed across the genome (Table S1).

**Figure 4:**
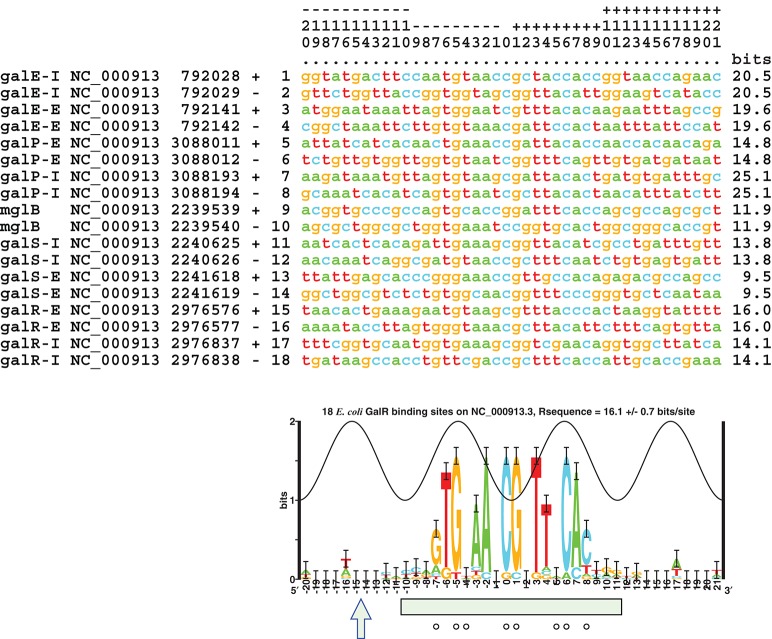
**Sequence alignment of 9 studied GalR binding sites (NC_000913.3) and corresponding sequence logo (Schneider and Stephens, 1990)**. The numbers on the top are to be read vertically. The peak of the sine wave indicates the location where the major groove of the DNA faces GalR (Papp et al., 1993). The information content of each site is indicated in the right hand column, they average to the area under the sequence logo, 16.1 ± 0.7 bits/site (Schneider et al., 1986; Schneider, 1997). A DNase I-hypersensitive site is indicated by an arrow and DNase I protection by a green box (Majumdar and Adhya, 1987). Bases protected from DMS by GalR are shown by circles (Majumdar and Adhya, 1989). The aligned listing and logo were created using Delila programs (Schneider et al., 1982, 1984). In this logo, base pair 9 in the consensus sequence mentioned elsewhere is marked as zero.

Further analysis of the original 9 GalR-target operators sequences with critical information content was conducted (Figure 1; Schneider and Mastronarde, 1996). A unique alignment of 42 bp length was obtained; the information content of the optimally aligned sites was R_sequence_ = 16.1 ± 0.7 bits/site for the 42 bp sequence range (Shannon, 1948; Pierce, 1980; Schneider et al., 1986). The information content needed to find these 9 sites in the 4,641,652 bp *E. coli* genome (NC_000913.3) is R_frequency_ = 18.98 bits/site; the information content in the sites is not sufficient for them to be found in the genome, R_sequence_/R_frequency_ = 0.85 ± 0.04, so the binding sites do not have enough information content for them to be located in the genome (Schneider et al., 1986; Schneider, 2000). This result implies that there could be 66 ± 32 sites in the genome. As shown in Figure 4, the sequence logo of the binding sites covers the DNase I protection segment (Majumdar and Adhya, 1987; Schneider and Stephens, 1990). There may be additional conservation near a DNase I-hypersensitive site in a major groove one helical turn from the central two major grooves bound by GalR (−16 and +17; Figure 4). The sequence conservation in the center of the site at bases 0 and 1 exceeds the sine wave, indicating that GalR binds to non-B-form DNA (Schneider, 2001) as was previously suggested (Majumdar and Adhya, 1989). An individual information weight matrix corresponding to positions −20 to +21 of the logo in Figure 4 was created and scanned across the *E. coli* genome (Schneider, 1997). Sixty sites were identified that contain more than 9.4 bits, the lowest information content of the biochemically proven sites. The sequences of novel GalR predicted sites corresponding to the logo are summarized in Table 2. R_frequency_ for these sites in the genome is 16.24 bits/site, which is close to the observed 16.3 ± 0.1 bits/site from all the predicted genomic sites.

**Table 2:** **GalR binding sites predicted by information theory**.

**Chromosome coordinate**	**GalR binding bits**	**Cognate gene**
37821	10.03857	*caiC*
43400	9.814199	*fixB*
**74447**	**11.21879**	***thiQ-thiB***
89735	11.40643	*mraZ*
**103352**	**9.751084**	***ftsQ***
**161073**	**9.635808**	***sfsA***
167231	9.801062	*mrcB*
**234579**	**10.76336**	***gloB***
**306553**	**10.13984**	***ecpE-ecpC***
**390979**	**9.664174**	-
**741888**	**11.21261**	***dtpD***
787535	12.30803	*gpmA*
791362	10.91125	*galE*
**792028**	**20.50652**	***galE***
**792141**	**19.60408**	***galE-modF***
914977	9.870273	*ybjE-aqpZ*
986589	10.22057	*ompF*
**1109183**	**10.44796**	***opgC-opgG***
1191794	9.613439	*purB*
1253804	9.870957	*ycgV*
1307943	10.76489	*clsA*
**1347064**	**9.781962**	***rnb***
1353246	10.53007	*sapD*
**1466984**	**9.647879**	-
1539818	12.88323	*narY-narU*
1572923	11.06094	*pqqL*
1712019	10.58487	*rsxE-dtpA*
1798963	9.813867	*pheS-pheM*
1803105	9.629957	-
**1857739**	**13.26586**	***ydjI***
1958198	11.84791	*torY-cutC*
2012349	10.87027	-
**2076502**	**11.88679**	***yeeR-yeeT***
2188111	11.89384	*yehA*
**2239539**	**11.87394**	***mglB***
**2240625**	**13.75395**	***mglB-galS***
**2241618**	**9.470329**	***galS***
**2241771**	**10.95166**	***galS-yeiB***
2390045	11.86583	*yfbP-nuoN*
2585453	9.76911	*aegA*
**2738463**	**10.28531**	***pheA***
2751444	11.95696	*nadK*
2783823	12.3442	*ypjC*
2839356	12.65853	*ascG-ascF*
**2976576**	**16.03935**	***omrB-galR***
**2976837**	**14.07919**	***galR***
3069624	11.39436	*mscS*
**3088011**	**14.78781**	***metK-galP***
**3088193**	**25.1484**	***metK-galP***
3115470	9.845925	*sslE*
3236977	10.89744	*ygjQ*
3287545	12.34189	*yraH*
3288641	10.85878	*yraI*
3492468	9.731226	*ppiA-tsgA*
**3656067**	**10.56542**	***hdeB***
3665637	9.573374	*gadX*
3700787	10.21665	*yhjV*
**4124542**	**13.28446**	***cytR-priA***
4155030	10.04465	*argC*
4573916	11.67178	-

*The bold are also present in Table S1*.

### Functional analysis of the putative GalR binding sites using ChIP-chip assays

**Figure 5:**
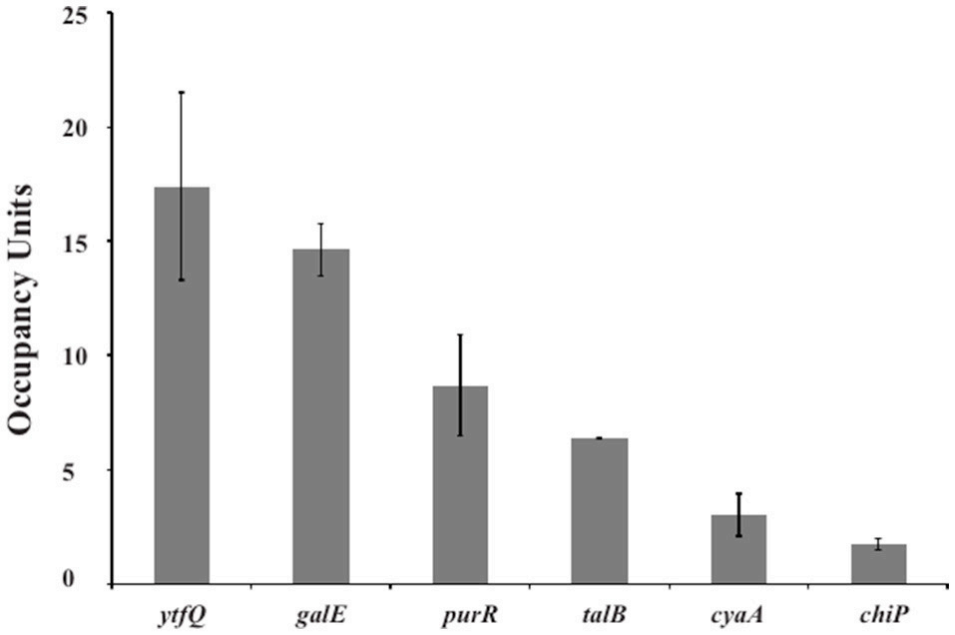
**Validation of GalR binding for selected regions identified by ChIP-chip**. ChIP-qPCR measurements of GalR association with regions identified as putative GalR-bound regions by ChIP-chip, upstream of the indicated genes. Occupancy units represent background-subtracted enrichment relative to a control region within the transcriptionally inert *bglB* gene. Error bars indicate one standard deviation from the mean (*n* = 3).

For the functional analysis of the putative binding sites, a ChIP-chip assay was performed to detect GalR target sequences genome-wide *in vivo* (Collas, 2010; Wade, 2015). In this ChIP-chip assay the binding of C-terminally TAP (tandem affinity purification) -tagged GalR (tagged at its native locus in an unmarked strain) was mapped across the *E. coli* genome. The experimental data resulting from ChIP-chip analysis were validated by quantitative real-time PCR (ChIP/qPCR). To demonstrate that the ChIP signal was not an artifact of the TAP tag, we constructed an unmarked derivative of *E. coli* MG1655 that expressed a C-terminally FLAG_3_-tagged GalR from its native locus. We selected six (*ytfQ, galE, purR, talB, cyaA*, and *chiP*) sites for validation, including *ytfQ, talB*, and *cyaA* that had not been described or predicted previously. In all cases, we detected significant signal of GalR binding indicating that these are genuine sites of GalR binding (Figure 5). The inferred binding sites from ChIP-chip assays are listed in Table 3. We identified 15 GalR-bound regions, four of which contain two operators. These include 8 known operators (in *galE, galP, galS, galR, chip*, and *mglB*; Weickert and Adhya, 1993b; Plumbridge et al., 2014). Thirteen of the 15 putative GalR-bound regions overlap an intergenic region upstream of a gene start. This is a strong enrichment over the number expected by chance (only ~12% of the genome is intergenic).

**Table 3:** **GalR-bound regions identified by ChIP-chip assays**.

**Peak probe position^a^**	**Binding score^b^**	**Nearby gene(s)^c^**	**Inferred binding site(s)^d^**
**8005**	**15.4**	**yaaJ/talB**	**TTGGTAACGTTTACA**
708069	4.9	chiP	ATGAAAGCGGTTACA
767563	4.5	(mngA)	GTGGAAGCGGTTACG
**792029**	**78.1**	**galE**	**GTGGTAGCGGTTACA**
792441			GTGGAATCGTTTACA
1627951	3.9	(ydfG)	GTGGTAACGTTTACG
**1737880**	**9.5**	**ynhF/purR**	**GTGGAAACGTTTGCT**
1737791			AGGCAAACGTTTACC
**2240625**	**9.5**	**mglB**	**TTGAAAGCGGTTACA**
**2241771**	**10.9**	**galS**	**ATGGAAACGGTTACA**
**2241619**			**GTGGCAACGGTTTCC**
**2976577**	**21.2**	**omrB/galR**	**GGGTAAACGCTTACA**
**3088194**	**115.4**	**galP**	**GTGTAATCGCTTACA**
3532886	4.6	yhgE/pck	ATGATATCGTTTACA
3991055	12.4	hemC/cyaA	GTGGTAACGGTTACC
4124542	3.5	cytR	GTGAAAACGGTTACA
4338179	6.9	adiY	ATGGCAACGTTTTCA
4338257			GTGGTTACGCTTTCA
**4449971**	**22**	**ppa/ytfQ**	**GTGGAAACGCTTACT**

*The bold labeled motifs are the GRS as defined in text*.

a *Genome coordinate corresponding to the center of the microarray probe in the associated GalR-bound region*.

b *Ratio of ChIP-chip signal for the ChIP and input control samples, for the peak probe (i.e., the microarray probe with the highest ratio in the GalR-bound region)*.

c *Genes in parentheses correspond to peak probes whole genomic location does not overlap with an intergenic region upstream of a gene. All other genes listed begin immediately downstream of intergenic regions that overlap the peak probe*.

d *Putative GalR binding site(s) identified using MEME*.

### Global transcription profile in the presence and absence of GalR

**Figure 6:**
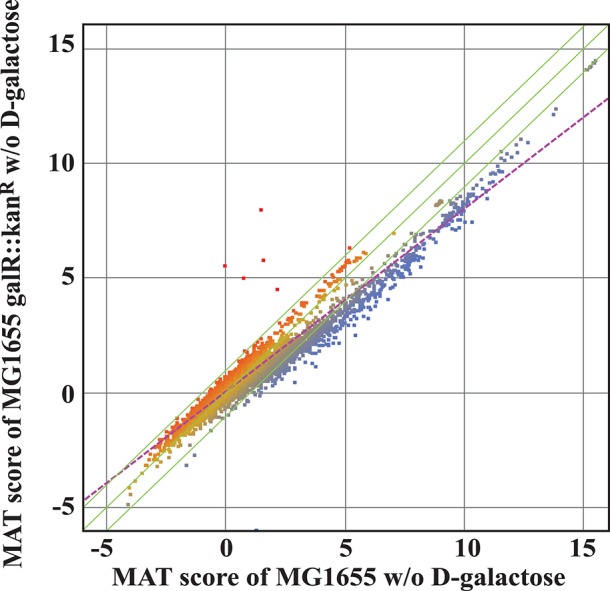
**MAT analysis of the transcriptome of wild type and Δ***galR*** cells grown in M63 minimal medium**. Green lines represent the mean ± 2SD, while the purple dotted line represents the regression line. The red represents the up genes and the blue represents the down genes. There is a marked down-regulation of many genes in the absence of GalR.

Since both *in silico* investigation and ChIP-chip assays suggested that the regulatory role of GalR goes beyond D-galactose metabolism, we used transcriptome profiling to gain further insight into the impact of GalR on genome-wide transcription. To evaluate the effect of *galR* deletion on global gene expression patterns, we compared the ratio of RNA isolated from a Δ*galR* mutant to that isolated from wild-type cells, using DNA tiling microarrays (Tokeson et al., 1991). The results of the transcriptional analysis are displayed in the MAT plot shown in Figure 6. For all analysis, we arbitrarily selected a stringent ratio cut-off of 3. We identified 238 genes with values exceeding this cut-off (Table S2). These 238 genes are transcribed from 158 promoters. Three transcripts (5 genes) of the 158 promoters are up-regulated (GalR acting as a repressor) and 155 transcripts (233 genes) are down-regulated (GalR acting as an activator; Table S2). Interestingly, several genes including *mglB* are dys-regulated by GalR but fall outside of the cut-off range. All three (*galP, galP1*, and *galP2*) of the up-regulated promoters have adjacent operators. Of the 155 down-regulated promoters, 4 promoters contain adjacent operators and the remaining 151 do not.

## Discussion

Using a combination of bioinformatic and experimental approaches we identified many putative novel GalR operators in the *E. coli* genome. As expected, several of these putative operators were identified by both information theory and ChIP-chip assays, demonstrating that they represent genuine GalR binding sites. Thus, we have substantially expanded the known GalR regulon. Surprisingly, our data suggest that GalR, a regulator of D-galactose metabolism, also regulates the expression of genes involved in other cellular processes. Interestingly, three of the putative novel GalR target genes—*cytR, purR*, and *adiY*—encode transcription factors, suggesting that GalR may be part of a more complex regulatory network. Moreover, putative GalR operators upstream of *cytR* and *purR* overlap with operators for CytR and PurR, respectively, indicating combinatorial regulation of these genes (Meng et al., 1990; Rolfes and Zalkin, 1990; Mengeritsky et al., 1993). Despite our identification of GalR operators with high confidence upstream of genes mentioned above, our expression microarray data show little or no regulation of these genes by GalR. We propose that regulation of these genes by GalR is condition-specific, requiring input from additional regulatory factors.

### Role of GalR in gene regulation

**Figure 7:**
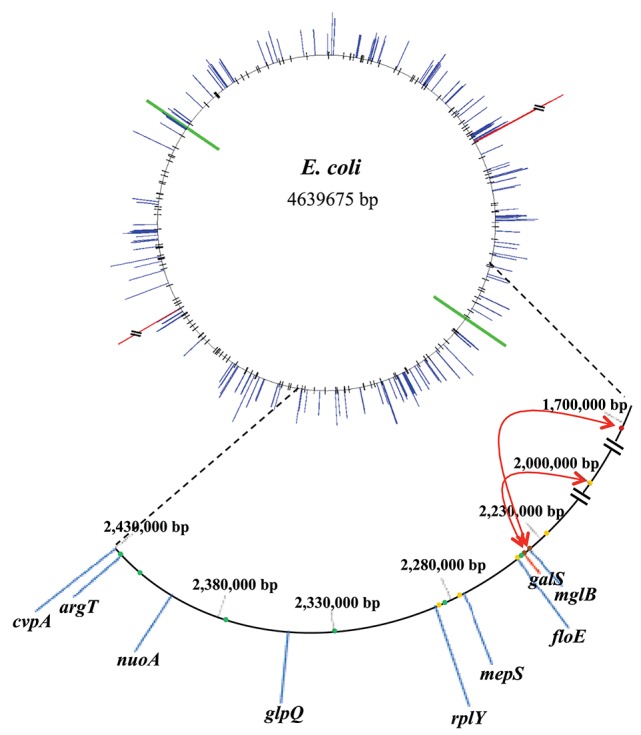
**Correlation of GalR operator locations and change in transcription pattern in the absence of GalR relative to in the wild type ***E. coli*** in the bacterial genome**. The *ori* and *ter* of replication are shown by green lines. Blue lines indicate the extent of down-regulated genes while red lines indicate the extent of up-regulated genes. The 165 GalR operators, demonstrable or potential, are shown as black lines in the top part. In the enlarged part (from 1.7 to 2.43 Mb), the extent of down-regulated and up-regulated genes are shown in blue and red lines, respectively. The dots represent some of the GalR operators. GRS and CAS are shown as green and orange dots, respectively while brown dots indicate that the binding sites serve as both GRS and CAS. The red arrows display the interactions between GalR operators detected by 3C assays.

DNA tiling array analysis revealed that the transcription of a surprisingly large number of promoters (158) in *E. coli* is dys-regulated by deletion of the *galR* gene. On the other hand, we identified 165 established or potential GalR operators in the chromosome, 76 of which are located between −200 to +400 bp from the *tsp* of promoters (cognate), and the other 89 operators are not (Table S1). We called the former group of operators, “Gene Regulatory Sites” (GRS, listed in Table 4). Consistent with a previous proposal (Macvanin and Adhya, 2012), we believe that 89 non-cognate operators around the chromosome are playing an architectural role in chromosome organization. The unattached operators would be referred to as “Chromosome Anchoring Sites” (CAS). Some of the sites may serve as both GRS and CAS. The 76 (46%) GRS and 89 (54%) CAS are shown in Table S1. Seventy-six GRS include 9 previously known operators of the *gal* regulon (see Figure 1); the other 67, which control promoters, were not known previously. The discovery of new GRS indicates that GalR, a well-known regulator of D-galactose metabolism, also regulates the expression of other genes. Among the new GRS, 3 (in *yaaJ, purR*, and *ytfQ* promoters) were confirmed by *in vivo* DNA-binding (ChIP-chip assays) as shown in Table 3. The salient features of our findings presented in this paper are shown schematically in Figure 7.

**Table 4:** **Gene Regulatory Sites discovered by sequence analysis**.

**Operator sequence**	**Strand**	**Chromosome**	**Coordinate**	**Cognate gene**
TTGGTAACGTTTACAC	−	7997	8012	*yaaJ/b0007*
GTTTAACCGCATTCAC	−	10286	10301	*satP/b0010*
GCGCAACCGCTACCAC	+	74440	74455	*thiP/b0067*
GTGGTGGCGCTTACAC	+	74460	74475	*thiP/b0067*
GTGAACGCCATTACAC	+	103345	103360	*ftsQ/b0093*
CTGAAAGGGTTTGCAC	+	118258	118273	*nadC/b0109*
GTGAACTCTTTTCCAC	+	151361	151376	*yadK/b0136*
TTGCAAATGGTTCCAC	−	151873	151888	*yadL/b0137*
GTGAAAATGATTGCAC	−	155580	155595	*yadV/b0140*
GTGGAAAAGTTTCCAC	−	234572	234587	*gloB/b0212*
GTAGAAACGCCTGCAC	+	329207	329222	*betI/b0313*
GTGGCATCGTCTTCAC	−	397495	397510	*sbmA/0377*
GTTTAAGCACTTTCAC	+	684934	684949	*gltL/b0652*
ATGAAATCGATGCCAC	−	720300	720315	*speF/b0693*
**ATGTAACCGCTACCAC**	**+**	**792021**	**792036**	***galE/b0759***
**GTGGAATCGTTTACAC**	**+**	**792134**	**792149**	***galE/b0759***
GTGAAGGCGCTGTCAC	−	863678	863693	*fsaA/b0825*
GTAAACCCGGTTTCAC	−	957266	957281	*ycaP/b0906*
GTCAAAACAGTTGCAC	+	1074131	1074146	*rutR/b1013*
TTGCAACCGTTTTCAC	−	1109176	1109191	*opgG/b1048*
GTGACATCGCGTCCAC	−	1110864	1113406	*opgH/b1049*
GAGGAACCGGTAGCAC	−	1156743	1156758	*holB/b1099*
GTGCAAACGCTATCAG	−	1176651	1176666	*lolD/b1117*
CTGAAATGGCTTTCAC	+	1413858	1413873	*ralR/b1348*
CTGCAAGCGCTTGAAC	+	1674225	1674240	*tqsA/b1601*
GAGCAAACGTTTCCAC	+	1737872	1737887	*purR/b1658*
ATGGAAGCTTTTCCAC	+	1771431	1771446	*ydiM/b1690*
GAGTAACCGTCTACAC	−	1791263	1791278	*ydiU/b1706*
GGGAAAACGATGCCAC	−	1857732	1857747	*ydjI/b1773*
GTGTCATCGACTGCAC	−	1896369	1896384	*nudL/b1813*
GTGCAGGAGATTGCAC	+	2005842	2005857	*fliT/b1926*
ATGGAAACATTTACAC	+	2012342	2012357	*yedN/b1932*
GTGAAGAGGGTTTCAC	−	2076495	2076510	*yeeS/b2002*
ATGCAACCGGTTACCC	−	2077222	2077237	*cbeA/b2004*
GTGTACGCATTTCCAC	+	2108205	2108220	*glf/b2036*
GTGCACCGGATTTCAC	+	2239532	2239547	*mglB/b2150*
TTGAAAGCGGTTACAT	+	2240618	2240633	*galS/b2151*
GGGAAACCGTTGCCAC	+	2241611	2241626	*galS/b2151*
GCGGAATCGGTTCAAC	+	2278144	2278159	*yejG/b2181*
GTGCGAACTCTTCCAC	+	2414919	2414934	*pta/b2297*
**CTGCATCCGTTTGCAC**	**+**	**2427600**	**2427615**	***argT/b2310***
CTGCAATCGCCTTCAC	+	2527286	2527301	*yfeH/b2410*
ATGCAATCGGTTACGC	−	2634124	2634139	*guaB/b2508*
GTGTACTCTATTACAC	−	2637479	2637494	*bamB/b2512*
**GTAAAGACGATTTCAC**	**+**	**2661317**	**2661332**	***iscS/b2530***
GTGTCGCCGTTTTCAC	+	2796513	2796528	*ygaU/b2665*
GAGGAAGCGGTTCGAC	+	2817230	2817245	*yqaB/b2690*
CTGGAAGCGATTGCCC	−	2832047	2832064	*norR/b2709*
GTGTGAACATTTCCAC	−	2837945	2837960	*hydN/b2713*
AAGAAACCGGTTTCAC	−	2839425	2839440	*ascF/b2715*
CTGCAAGCCGTTGCAC	+	2848912	2848927	*hycC/b2723*
ATGTAAGCGTTTACCC	+	2976569	2976584	*galR/b2837*
GTTCGACCGCTTTCAC	−	2976830	2976845	*galR/b2837*
GTTAAAGCATTTACAC	−	2995106	2995121	*ygeK/b2856*
ATGCAAGTGCTTTCAC	−	3041236	3041251	*ygfZ/b2898*
**CTGAAACCGATTACAC**	**+**	**3088004**	**3088019**	***galP/b2943***
**GTGTAAGCGATTACAC**	**+**	**3088186**	**3088201**	***galP/b2943***
GTTGCAGCGATTTCAC	+	3133074	3133089	*yghR/b2984*
GAGGAAGTGATTGCAC	−	3320107	3320122	*yhbX/b3173*
CTGGAACCGTATTCAC	+	3372189	3372204	*nanT/b3224*
GTGGGATCGAGTACAC	−	3375005	3375020	*dcuD/b3227*
**GTAAGAACGGTTACAC**	**−**	**3453341**	**3453356**	***rpsJ/b3321***
AGGAAACCGCTTCCAC	−	3540370	3540385	*feoA/b3408*
CAGGAAGCGCTTTCAC	−	3552425	3552440	*malP/b3417*
ATCAAATCGATTACAC	−	3710451	3710466	*eptB/b3546*
GCGCAACGGCTTCCAC	+	3760922	3760937	*selA/b3591*
GCGAAATTGATTACAC	+	3824831	3824846	*trmH/b3651*
**GCGCAACCGTTCTCAC**	**+**	**3884368**	**3884383**	***rpmH/b3703***
GGGTAATCGCGTCCAC	−	4256787	4256802	*dgkA/b4042*
GTGCAAAAGATTGCAC	−	4281671	4281686	*yjcE/b4065*
GGGTAATCGGTTTTAC	−	4330520	4330535	*proP/b4111*
GAGAAAACGCTTCAAC	−	4378149	4378164	*ampC/b4150*
CTGGCATCGTTTACAC	−	4433627	4433642	*qorB/b4211*
AAGTAAGCGTTTCCAC	−	4449964	4449979	*ytfQ/b4227*
TTGCCACCGCTTTCAC	−	4483949	4483964	*holC/b4259*

*The motifs in bold letters are also present in Table S2*.

Although we identified 158 transcripts whose expression was regulated by GalR, very few of these are associated with a putative GalR operator identified *in silico* and/or ChIP-chip assays, strongly suggesting that the majority of regulation by GalR occurs indirectly. Based on our earlier observation that GalR mediates mega-loop formation, we propose that long-range oligomerization of GalR indirectly regulates transcription by altering chromosome structure. There are at least three possible mechanisms for such regulation: indirect control, enhancer activity, and modulation of DNA superhelicity. In the indirect control model, GalR directly regulates another regulator, such as PurR or CytR, and the downstream regulator directly regulates other genes. The regulation by GalR is indirect, but occurs by a classical regulatory mechanism. In the enhancer activity model, GalR stimulates transcription of some target genes by binding to a distal site and forming an enhancer-loop with a protein bound to the promoter region. Examples of enhancer activity have been described before for some prokaryotic and many eukaryotic promoters (Rombel et al., 1998; Schaffner, 2015). In the DNA superhelicity modulation model, GalR creates DNA topological domains by mega-loop formation and defines local chromosomal superhelicity by GalR-GalR interactions between distally bound dimers. The strength of a promoter is usually defined by superhelical nature of the DNA (Pruss and Drlica, 1989; Lim et al., 2003). We propose that GalR entraps different amount of superhelicity in different topological domains and thus controls transcription of the constituent promoters. In the absence of GalR such domains are not formed resulting in a change in local DNA superhelicity, and thus a change in the strength of the constituent promoters. In this model, GalR protein indirectly regulates gene transcription as an architectural protein. We are currently studying the regional superhelicities in the entire chromosome in the presence and absence of GalR as well as the implication of genes affected by GalR, but independent of D-galactose metabolism (Lal et al., 2016).

## Author contributions

ZQ: designed genome-wide sequence analysis, interpreted sequence analysis data and tiling array data; AT and SL: executed tiling array experiments and data analysis; XH: executed genome-wide sequence analysis; TD: integrated tiling array and genome-wide sequence data; AS and JW: executed ChIP-chip and ChIP-qPCR experiments and data analysis; DL: data analysis; TS: executed Information Theory and data analysis; SA: organized and designed experiments, and data analysis. All authors contributed to the manuscript preparation.

### Conflict of interest statement

The authors declare that the research was conducted in the absence of any commercial or financial relationships that could be construed as a potential conflict of interest.

## References

[B1] Adhya S. Miller W. (1979). Modulation of the two promoters of the galactose operon of *Escherichia coli*. Nature 279, 492–494. 10.1038/279492a0 221830

[B2] Aiba H. Adhya S. de Crombrugghe B. (1981). Evidence for two functional gal promoters in intact *Escherichia coli* cells. J. Biol. Chem. 256, 11905–11910. 6271763

[B3] Aki T. Adhya S. (1997). Repressor induced site-specific binding of HU for transcriptional regulation. EMBO J. 16, 3666–3674. 10.1093/emboj/16.12.3666 9218807 PMC1169990

[B4] Aki T. Choy H. E. Adhya S. (1996). Histone-like protein HU as a specific transcriptional regulator: co-factor role in repression of gal transcription by GAL repressor. Genes Cells 1, 179–188. 10.1046/j.1365-2443.1996.d01-236.x 9140062

[B5] Baba T. Ara T. Hasegawa M. Takai Y. Okumura Y. Baba M.. (2006). Construction of *Escherichia coli* K-12 in-frame, single-gene knockout mutants: the Keio collection. Mol. Syst. Biol. 2, 2006 02008. 10.1038/msb4100050 16738554 PMC1681482

[B6] Bailey T. L. Elkan C. (1994). Fitting a mixture model by expectation maximization to discover motifs in biopolymers. Proc. Int. Conf. Intell. Syst. Mol. Biol. 2, 28–36. 7584402

[B7] Butland G. Peregrín-Alvarez J. M. Li J. Yang W. Yang X. Canadien V.. (2005). Interaction network containing conserved and essential protein complexes in *Escherichia coli*. Nature 433, 531–537. 10.1038/nature03239 15690043

[B8] Choy H. E. Park S. W. Aki T. Parrack P. Fujita N. Ishihama A.. (1995). Repression and activation of transcription by Gal and Lac repressors: involvement of alpha subunit of RNA polymerase. EMBO J. 14, 4523–4529. 7556095 10.1002/j.1460-2075.1995.tb00131.x PMC394544

[B9] Collas P. (2010). The current state of chromatin immunoprecipitation. Mol. Biotechnol. 45, 87–100. 10.1007/s12033-009-9239-8 20077036

[B10] Datsenko K. A. Wanner B. L. (2000). One-step inactivation of chromosomal genes in *Escherichia coli* K-12 using PCR products. Proc. Natl. Acad. Sci. U.S.A. 97, 6640–6645. 10.1073/pnas.120163297 10829079 PMC18686

[B11] Dekker J. Rippe K. Dekker M. Kleckner N. (2002). Capturing chromosome conformation. Science 295, 1306–1311. 10.1126/science.1067799 11847345

[B12] Glasner J. D. Liss P. Plunkett G. III. Darling A. Prasad T.. (2003). ASAP, a systematic annotation package for community analysis of genomes. Nucleic Acids Res. 31, 147–151. 10.1093/nar/gkg125 12519969 PMC165572

[B13] Hogg R. W. Voelker C. Von Carlowitz I. (1991). Nucleotide sequence and analysis of the mgl operon of *Escherichia coli* K12. Mol. Gen. Genet. 229, 453–459. 10.1007/BF00267469 1719366

[B14] Irani M. H. Orosz L. Adhya S. (1983). A control element within a structural gene: the gal operon of *Escherichia coli*. Cell 32, 783–788. 10.1016/0092-8674(83)90064-8 6299576

[B15] Ishihama A. (2009). The nucleoid: an overview. EcoSal Plus 3, 1–47. 10.1128/ecosal.2.6 26443760

[B16] Johnson W. E. Li W. Meyer C. A. Gottardo R. Carroll J. S. Brown M.. (2006). Model-based analysis of tiling-arrays for ChIP-chip. Proc. Natl. Acad. Sci. U.S.A. 103, 12457–12462. 10.1073/pnas.0601180103 16895995 PMC1567901

[B17] Kar S. Adhya S. (2001). Recruitment of HU by piggyback: a special role of GalR in repressosome assembly. Genes Dev. 15, 2273–2281. 10.1101/gad.920301 11544184 PMC312769

[B18] Kar S. Edgar R. Adhya S. (2005). Nucleoid remodeling by an altered HU protein: reorganization of the transcription program. Proc. Natl. Acad. Sci. U.S.A. 102, 16397–16402. 10.1073/pnas.0508032102 16258062 PMC1283455

[B19] Lal A. Dhar A. Trostel A. Kouzine F. Seshasayee A. S. Adhya S. (2016). Genome scale patterns of supercoiling in a bacterial chromosome. Nat. Commun. 7:11055. 10.1038/ncomms11055 27025941 PMC4820846

[B20] Lee S. J. Trostel A. Adhya S. (2014). Metabolite changes signal genetic regulatory mechanisms for robust cell behavior. MBio 5:e00972-13. 10.1128/mBio.00972-13 24473130 PMC3903281

[B21] Lim H. M. Lewis D. E. Lee H. J. Liu M. Adhya S. (2003). Effect of varying the supercoiling of DNA on transcription and its regulation. Biochemistry 42, 10718–10725. 10.1021/bi030110t 12962496

[B22] Loferer-Krössbacher M. Klima J. Psenner R. (1998). Determination of bacterial cell dry mass by transmission electron microscopy and densitometric image analysis. Appl. Environ. Microbiol. 64, 688–694. 9464409 10.1128/aem.64.2.688-694.1998 PMC106103

[B23] Luijsterburg M. S. White M. F. van Driel R. Dame R. T. (2008). The major architects of chromatin: architectural proteins in bacteria, archaea and eukaryotes. Crit. Rev. Biochem. Mol. Biol. 43, 393–418. 10.1080/10409230802528488 19037758

[B24] Macvanin M. Adhya S. (2012). Architectural organization in *E. coli* nucleoid. Biochim. Biophys. Acta 1819, 830–835. 10.1016/j.bbagrm.2012.02.012 22387214 PMC7449586

[B25] Majumdar A. Adhya S. (1984). Demonstration of two operator elements in gal: *in vitro* repressor binding studies. Proc. Natl. Acad. Sci. U.S.A. 81, 6100–6104. 10.1073/pnas.81.19.6100 6385008 PMC391867

[B26] Majumdar A. Adhya S. (1987). Probing the structure of gal operator-repressor complexes. conformation change in DNA. J. Biol. Chem. 262, 13258–13262. 3308875

[B27] Majumdar A. Adhya S. (1989). Effect of ethylation of operator-phosphates on Gal repressor binding. DNA contortion by repressor. J. Mol. Biol. 208, 217–223. 10.1016/0022-2836(89)90383-5 2671389

[B28] Meng L. M. Kilstrup M. Nygaard P. (1990). Autoregulation of PurR repressor synthesis and involvement of purR in the regulation of purB, purC, purL, purMN and guaBA expression in *Escherichia coli*. Eur. J. Biochem. 187, 373–379. 10.1111/j.1432-1033.1990.tb15314.x 2404765

[B29] Mengeritsky G. Goldenberg D. Mendelson I. Giladi H. Oppenheim A. B. (1993). Genetic and biochemical analysis of the integration host factor of *Escherichia coli*. J. Mol. Biol. 231, 646–657. 10.1006/jmbi.1993.1316 8515442

[B30] Musso R. E. Di Lauro R. Adhya S. de Crombrugghe B. (1977). Dual control for transcription of the galactose operon by cyclic AMP and its receptor protein at two interspersed promoters. Cell 12, 847–854. 10.1016/0092-8674(77)90283-5 200371

[B31] Oberto J. Nabti S. Jooste V. Mignot H. Rouviere-Yaniv J. (2009). The HU regulon is composed of genes responding to anaerobiosis, acid stress, high osmolarity and SOS induction. PLoS ONE 4:e4367. 10.1371/journal.pone.0004367 19194530 PMC2634741

[B32] Oehler S. Müller-Hill B. (2010). High local concentration: a fundamental strategy of life. J. Mol. Biol. 395, 242–253. 10.1016/j.jmb.2009.10.056 19883663

[B33] Papp P. P. Chattoraj D. K. Schneider T. D. (1993). Information analysis of sequences that bind the replication initiator repa. J. Mol. Biol. 233, 219–230. 10.1006/jmbi.1993.1501 8377199

[B34] Pierce J. R. (1980). An Introduction to Information Theory: Symbols, Signals and Noise, 2nd Edn. New York, NY: Dover Publications, Inc.

[B35] Plumbridge J. Bossi L. Oberto J. Wade J. T. Figueroa-Bossi N. (2014). Interplay of transcriptional and small RNA-dependent control mechanisms regulates chitosugar uptake in *Escherichia coli* and Salmonella. Mol. Microbiol. 92, 648–658. 10.1111/mmi.12573 24593230

[B36] Priyadarshini R. Cugini C. Arndt A. Chen T. Tjokro N. O. Goodman S. D.. (2013). The nucleoid-associated protein HUbeta affects global gene expression in Porphyromonas gingivalis. Microbiology 159, 219–229. 10.1099/mic.0.061002-0 23175503 PMC3709559

[B37] Pruss G. J. Drlica K. (1989). DNA supercoiling and prokaryotic transcription. Cell 56, 521–523. 10.1016/0092-8674(89)90574-6 2645054

[B38] Qian Z. Dimitriadis E. K. Edgar R. Eswaramoorthy P. Adhya S. (2012). Galactose repressor mediated intersegmental chromosomal connections in *Escherichia coli*. Proc. Natl. Acad. Sci. U.S.A. 109, 11336–11341. 10.1073/pnas.1208595109 22733746 PMC3396475

[B39] Rolfes R. J. Zalkin H. (1990). Autoregulation of *Escherichia coli* purR requires two control sites downstream of the promoter. J. Bacteriol. 172, 5758–5766. 2211510 10.1128/jb.172.10.5758-5766.1990 PMC526892

[B40] Rombel I. North A. Hwang I. Wyman C. Kustu S. (1998). The bacterial enhancer-binding protein NtrC as a molecular machine. Cold Spring Harb. Symp. Quant. Biol. 63, 157–166. 10.1101/sqb.1998.63.157 10384279

[B41] Roy S. Dimitriadis E. K. Kar S. Geanacopoulos M. Lewis M. S. Adhya S. (2005). Gal repressor-operator-HU ternary complex: pathway of repressosome formation. Biochemistry 44, 5373–5380. 10.1021/bi047720t 15807530

[B42] Roy S. Semsey S. Liu M. Gussin G. N. Adhya S. (2004). GalR represses galP1 by inhibiting the rate-determining open complex formation through RNA polymerase contact: a GalR negative control mutant. J. Mol. Biol. 344, 609–618. 10.1016/j.jmb.2004.09.070 15533432

[B43] Schaffner W. (2015). Enhancers, enhancers - from their discovery to today's universe of transcription enhancers. Biol. Chem. 396, 311–327. 10.1515/hsz-2014-0303 25719310

[B44] Schneider T. D. (1997). Information content of individual genetic sequences. J. Theor. Biol. 189, 427–441. 10.1006/jtbi.1997.0540 9446751

[B45] Schneider T. D. (2000). Evolution of biological information. Nucleic Acids Res. 28, 2794–2799. 10.1093/nar/28.14.2794 10908337 PMC102656

[B46] Schneider T. D. (2001). Strong minor groove base conservation in sequence logos implies DNA distortion or base flipping during replication and transcription initiation. Nucleic Acids Res. 29, 4881–4891. 10.1093/nar/29.23.4881 11726698 PMC96701

[B47] Schneider T. D. Mastronarde D. N. (1996). Fast multiple alignment of ungapped DNA sequences using information theory and a relaxation method. Discrete Appl. Math. 71, 259–268. 10.1016/S0166-218X(96)00068-6 19953199 PMC2785095

[B48] Schneider T. D. Stephens R. M. (1990). Sequence logos - a new way to display consensus sequences. Nucleic Acids Res. 18, 6097–6100. 10.1093/nar/18.20.6097 2172928 PMC332411

[B49] Schneider T. D. Stormo G. D. Gold L. Ehrenfeucht A. (1986). Information-content of binding-sites on nucleotide-sequences. J. Mol. Biol. 188, 415–431. 10.1016/0022-2836(86)90165-8 3525846

[B50] Schneider T. D. Stormo G. D. Haemer J. S. Gold L. (1982). A design for computer nucleic-acid-sequence storage, retrieval, and manipulation. Nucleic Acids Res. 10, 3013–3024. 10.1093/nar/10.9.3013 7099972 PMC320671

[B51] Schneider T. D. Stormo G. D. Yarus M. A. Gold L. (1984). Delila system tools. Nucleic Acids Res. 12, 129–140. 10.1093/nar/12.1Part1.129 6694897 PMC320990

[B52] Semsey S. Geanacopoulos M. Lewis D. E. Adhya S. (2002). Operator-bound GalR dimers close DNA loops by direct interaction: tetramerization and inducer binding. EMBO J. 21, 4349–4356. 10.1093/emboj/cdf431 12169637 PMC126169

[B53] Shannon C. E. (1948). A mathematical theory of communication. Bell Syst. Tech. J. 27, 379–423. 10.1002/j.1538-7305.1948.tb01338.x

[B54] Skoko D. Yoo D. Bai H. Schnurr B. Yan J. McLeod S. M.. (2006). Mechanism of chromosome compaction and looping by the *Escherichia coli* nucleoid protein Fis. J. Mol. Biol. 364, 777–798. 10.1016/j.jmb.2006.09.043 17045294 PMC1988847

[B55] Stringer A. M. Currenti S. A. Bonocora R. P. Petrone B. L. Palumbo M. J. Reilly A. E.. (2014). Genome-scale analyses of *Escherichia coli* and *Salmonella enterica* AraC reveal non-canonical targets and an expanded core regulon. J. Bacteriol. 196, 660–671. 10.1128/JB.01007-13 24272778 PMC3911152

[B56] Stringer A. M. Singh N. Yermakova A. Petrone B. L. Amarasinghe J. J. Reyes-Diaz L.. (2012). FRUIT, a scar-free system for targeted chromosomal mutagenesis, epitope tagging, and promoter replacement in *Escherichia coli* and *Salmonella enterica*. PLoS ONE 7:e44841. 10.1371/journal.pone.0044841 23028641 PMC3459970

[B57] Tokeson J. P. Garges S. Adhya S. (1991). Further inducibility of a constitutive system: ultrainduction of the gal operon. J. Bacteriol. 173, 2319–2327. 2007555 10.1128/jb.173.7.2319-2327.1991 PMC207785

[B58] Wade J. T. (2015). Mapping transcription regulatory networks with ChIP-seq and RNA-seq. Adv. Exp. Med. Biol. 883, 119–134. 10.1007/978-3-319-23603-2_7 26621465

[B59] Weickert M. J. Adhya S. (1993a). Control of transcription of gal repressor and isorepressor genes in *Escherichia coli*. J. Bacteriol. 175, 251–258. 8416900 10.1128/jb.175.1.251-258.1993 PMC196120

[B60] Weickert M. J. Adhya S. (1993b). The galactose regulon of *Escherichia coli*. Mol. Microbiol. 10, 245–251. 7934815 10.1111/j.1365-2958.1993.tb01950.x

