# Supplementary material for: Genome-Wide Transcriptional Regulation and Chromosome Structural Arrangement by GalR in E. coli
Source: Front Mol Biosci. 2016 Nov 16;3:74. doi: 10.3389/fmolb.2016.00074 (PMC5110547; doi:10.3389/fmolb.2016.00074)
Supplement: Supplementary file 1 [file Table1.PDF]

Table S1: GalR Binding Operators discovered by whole genome search with conserved motif (Accession No. NC_000193.3)

Operator sequence	Strand	Chromosome coordinate		Cognate gene
**TTGGTAACGTTTACAC**	**-**	**7997**	**8012**	***yaaJ***
**GTTTAACCGCATTCAC**	**-**	**10286**	**10301**	***satP***
**GCGCAACCGCTACCAC**	**+**	**74440**	**74455**	***thiP***
**GTGGTGGCGCTTACAC**	**+**	**74460**	**74475**	***thiP***
**GTGAACGCCATTACAC**	**+**	**103345**	**103360**	***ftsQ***
GCGTAAGCGATATCAC	-	104703	104718	*ftsA*
**CTGAAAGGGTTTGCAC**	**+**	**118258**	**118273**	***nadC***
**GTGAACTCTTTTCCAC**	**+**	**151361**	**151376**	***yadK***
**TTGCAAATGGTTCCAC**	**-**	**151873**	**151888**	***yadL***
**GTGAAAATGATTGCAC**	**-**	**155580**	**155595**	***yadV***
ACGTAACCGATTTCAC	+	161066	161081	*sfsA*
TTGCCAGCGGTTGCAC	-	183465	183480	*cdaR*
GGGTAACCGTTTCTAC	+	198956	198971	*bamA*
**GTGGAAAAGTTTCCAC**	**-**	**234572**	**234587**	***gloB***
GTGCCGCCGATTGCAC	+	307873	307888	*ecpC*
**GTAGAAACGCCTGCAC**	**+**	**329207**	**329222**	***betI***
CTGTAAAAGATTCCAC	+	390972	390987	*yaiT*
**GTGAAGACGATGCCAC**	**-**	**397495**	**397510**	***sbmA***
CTGTCAGCGATTGCAC	+	415087	415102	*sbcC*
GGGAAAGCGATTTCAT	-	451144	451159	*cyoA*
GTGAAACCGTTGGCAG	-	463340	463355	*ppiD*
GTGAAAATGGTTGTAC	-	536809	536824	*glxR*
GTGAAAGCTATTGCCC	-	613520	613535	*fes*
GTGCAATCGTATGGAC	-	614945	614960	*entF*
CTTTAAACGATTCCAC	+	632394	632409	*ybdH*
GTGTAAACGGTGCCAG	-	646112	646127	*citT*
**GTTTAAGCACTTTCAC**	**+**	**684934**	**684949**	***gltL***
ATGTAACCGCTTTCAT	+	708061	708076	*chiP*
**GTGGCATCGATTTCAT**	**-**	**720300**	**720315**	***speF***
GTGCAGGCGTTGCCAC	-	741881	741896	*dtpD*
CTGAAATCGGTTTCGC	+	751457	751472	*ybgQ*
CCGTAACCGCTTCCAC	+	767566	767581	*mngA*
**ATGTAACCGCTACCAC**	**+**	**792021**	**792036**	***galE***
**GTGGAATCGTTTACAC**	**+**	**792134**	**792149**	***galE***
**GTGAAGGCGCTGTCAC**	**-**	**863678**	**863693**	***fsaA***
GTGTAATTGGTTCGAC	-	924213	924228	*clpA*
GTGAAATCGGTAGTAC	-	942073	942088	*dmsA*

Table S1 continued

**GTGAAACCGGGTTTAC**	**-**	**957266**	**957281**	***ycaP***
GTGTGAGCGTTTGCAA	-	980591	980606	*mukB*
**GTCAAAACAGTTGCAC**	**+**	**1074131**	**1074146**	***rutR***
GTGGAACCACTTTCAG	-	1081978	1081993	*efeU*
**GTGAAAACGGTTGCAA**	**-**	**1109176**	**1109191**	***opgG***
**GTGGACGCGATGTCAC**	**-**	**1111182**	**1111197**	***opgH***
**GTGCTACCGGTTCCTC**	**-**	**1156743**	**1156758**	***holB***
**GTGCAAACGCTATCAG**	**-**	**1176651**	**1176666**	***lolD***
CTGGAAGAGTTTGCAC	+	1231894	1231909	*umuC*
GTGGCATCTTTTGCAC	+	1297685	1297700	*adhE*
TTGTAAACCGTTTCAC	+	1347057	1347072	*rnb*
CGGTAATCGGTTTCAC	+	1354999	1355014	*sapB*
**CTGAAATGGCTTTCAC**	**+**	**1413858**	**1413873**	***ralR***
GTGAATTTGGTTCCAC	+	1456340	1456355	*paaD*
ATGGAAAAGATTCCAC	+	1466977	1466992	*ydbA*
GTGAAAGTGGTTAAAC	-	1501637	1501652	*tehB*
GTGAAAACGGTGAGAC	-	1524383	1524398	*yncE*
GTGTGATCGGTTACGC	-	1567912	1567927	*yddW*
GTGTAATCTCCTGCAC	-	1595793	1595808	*yneO*
**CTGCAAGCGCTTGAAC**	**+**	**1674225**	**1674240**	***tqsA***
GTGAAAGCCGTTTCTC	-	1678583	1678598	*ydgH*
GGGCAATCGGCTGCAC	-	1684964	1684979	*tus*
**GAGCAAACGTTTCCAC**	**+**	**1737872**	**1737887**	***purR***
**ATGGAAGCTTTTCCAC**	**+**	**1771431**	**1771446**	***ydiM***
**GAGTAACCGTCTACAC**	**-**	**1791263**	**1791278**	***ydiU***
ATGAAAGCGGATTCAC	+	1843179	1843194	*gdhA*
**GGGAAAACGATGCCAC**	**-**	**1857732**	**1857747**	***ydjI***
**GTGTCATCGACTGCAC**	**-**	**1896369**	**1896384**	***nudL***
CTGTAACCGTTGGCAC	-	1953258	1953273	*cmoA*
CTGGAACCGATCTCAC	-	1957474	1957489	*torY*
**GTGCAGGAGATTGCAC**	**+**	**2005842**	**2005857**	***fliT***
**ATGGAAACATTTACAC**	**+**	**2012342**	**2012357**	***yedN***
**GTGAAGAGGGTTTCAC**	**-**	**2076495**	**2076510**	***yeeS***
**ATGCAACCGGTTACCC**	**-**	**2077222**	**2077237**	***cbeA***
**GTGTACGCATTTCCAC**	**+**	**2108205**	**2108220**	***glf***
ATGGACTCGTTTTCAC	+	2123220	2123235	*cpsB*
GTGCCTTCGGTTTCAC	-	2154533	2154548	*mdtA*
GTAAAGCCGATTTCAC	-	2156575	2156590	*mdtB*
GTCCGACCGTTTTCAC	+	2221572	2221587	*bglX*
GTGCACCGGATTTCAC	+	2239532	2239547	*mglB*
TTGAAAGCGGTTACAT	+	2240618	2240633	*galS*

Table S1 continued

GGGAAACCGTTGCCAC	+	2241611	2241626	*galS*
ATGGAAACGGTTACAG	+	2241764	2241779	*yeiB*
GTCAAAGCCATTGCAC	+	2272841	2272856	*yejA*
**GCGGAATCGGTTCAAC**	**+**	**2278144**	**2278159**	***yejG***
GTGACAACCATTGCAC	-	2279322	2279337	*bcr*
**GTTCACTCGCTTCCAC**	**+**	**2326164**	**2326179**	***atoB***
**GGGCAACTGGTTTCAC**	**-**	**2374031**	**2374046**	***menE***
**GTGCGAACTCTTCCAC**	**+**	**2414919**	**2414934**	***pta***
**CTGCATCCGTTTGCAC**	**+**	**2427600**	**2427615**	***argT***
TTGAAAGCGTTTTCTC	+	2449479	2449494	*yfcO*
GTGCAGCCAATTGCAC	-	2479024	2479039	*dsdX*
GTGGAGGCGATTTCAC	+	2486615	2486630	*evgS*
CTGCAAACGCTTTAAC	+	2515865	2515880	*yfeA*
**CTGCAATCGCCTTCAC**	**+**	**2527286**	**2527301**	***yfeH***
**ATGCAATCGGTTACGC**	**-**	**2634124**	**2634139**	***guaB***
**GTGTACTCTATTACAC**	**-**	**2637479**	**2637494**	***bamB***
**GTAAAGACGATTTCAC**	**+**	**2661317**	**2661332**	***iscS***
CTGTCAGCGATTTCAC	+	2684933	2684948	*glyA*
TTGAAATCGTTTGCAT	+	2687476	2687491	*glrR*
ATGGAAAAGGTTGCAC	+	2738456	2738471	*pheA*
ATGAAAGCGAGTCCAC	-	2778946	2778961	*ypjA*
**GTGTCGCCGTTTTCAC**	**+**	**2796513**	**2796528**	***ygaU***
**GAGGAAGCGGTTCGAC**	**+**	**2817230**	**2817245**	***yqaB***
**CTGGAAGCGATTGCCC**	**-**	**2832049**	**2832064**	***norR***
**GTGTGAACATTTCCAC**	**-**	**2837945**	**2837960**	***hydN***
**AAGAAACCGGTTTCAC**	**-**	**2839425**	**2839440**	***ascF***
**CTGCAAGCCGTTGCAC**	**+**	**2848912**	**2848927**	***hycC***
GTGAAAAAGGTTACAC	-	2899003	2899018	*ygcU*
GTGAAGTGGATTCCAC	-	2922333	2922348	*yqcA*
GTTCACCCGCTTCCAC	-	2938098	2938113	*fucK*
TTGAAAATGGTTTCAC	+	2940333	2940348	*rlmM*
**ATGTAAGCGTTTACCC**	**+**	**2976569**	**2976584**	***galR***
**GTTCGACCGCTTTCAC**	**-**	**2976830**	**2976845**	***galR***
**GTTAAAGCATTTACAC**	**-**	**2995106**	**2995121**	***ygeK***
GAGAATGCGATTGCAC	-	3014388	3014403	*yqeC*
**ATGCAAGTGCTTTCAC**	**-**	**3041236**	**3041251**	***ygfZ***
ATGCAGCCGTTTTCAC	+	3054116	3054131	*pepP*
GTGCAGCTGGTTACAC	-	3058313	3058328	*serA*
**CTGAAACCGATTACAC**	**+**	**3088004**	**3088019**	***galP***
**GTGTAAGCGATTACAC**	**+**	**3088186**	**3088201**	***galP***
GAGTAACAGCTTGCAC	+	3107686	3107701	*speC*

Table S1 continued

GAGTAAACGCTTCGAC	-	3118507	3118522	*sslE*
**GTTGCAGCGATTTCAC**	**+**	**3133074**	**3133089**	***yghR***
GCGCAAACGCTGGCAC	-	3199317	3199332	*glnE*
CTGGAATAGCTTGCAC	-	3275969	3275984	*garD*
ATAAAAGCGATTTCAC	-	3295033	3295048	*lpoA*
**GAGGAAGTGATTGCAC**	**-**	**3320107**	**3320122**	***yhbX***
GTCGCACCGTTTGCAC	+	3323514	3323529	*glmM*
CTGCAATCGCTTTCGC	+	3326317	3326332	*ftsH*
CTGAAATCGATTTCTC	-	3337288	3337303	*mlaC*
AAGCAATCGCTTCCAC	+	3369816	3369831	*nanK*
**CTGGAACCGTATTCAC**	**+**	**3372189**	**3372204**	***nanT***
**GTGGGATCGAGTACAC**	**-**	**3375005**	**3375020**	***dcuD***
CTGCTAACGGTTTCAC	+	3385106	3385121	*argR*
**GTAAGAACGGTTACAC**	**-**	**3453341**	**3453356**	***rpsJ***
CTGAAAACGCTTCCCC	-	3517964	3517979	*aroB*
**AGGAAACCGCTTCCAC**	**-**	**3540370**	**3540385**	***feoA***
**CAGGAAGCGCTTTCAC**	**-**	**3552425**	**3552440**	***malP***
CTGAAAACGACTTCAC	-	3588616	3588631	*ugpC*
GTTCAATCATTTGCAC	-	3608455	3608470	*zntA*
GCGGAACCGCTTTTAC	+	3610731	3610746	*yhhS*
ATGAAACCGATCTCAC	-	3656060	3656075	*hdeB*
CTGTATGCGATTTCAC	+	3663033	3663048	*mdtF*
GTAGCAGCGGTTTCAC	+	3691548	3691563	*bcsB*
**ATCAAATCGATTACAC**	**-**	**3710451**	**3710466**	***eptB***
**GCGCAACGGCTTCCAC**	**+**	**3760922**	**3760937**	***selA***
TTGCAAGCGGTGACAC	+	3780373	3780388	*lldD*
CTTCAACCGCTTTCAC	-	3786691	3786706	*gpmM*
**GCGAAATTGATTACAC**	**+**	**3824831**	**3824846**	***trmH***
**GCGCAACCGTTCTCAC**	**+**	**3884368**	**3884383**	***rpmH***
GTGTGAACCTTTTCAC	-	3947822	3947837	*hdfR*
TTGTGATCGCTTTCAC	-	4058286	4058301	*typA*
ATGTGACCGATTACAC	-	4116669	4116684	*glpK*
GTTGCACCGTTTCCAC	-	4120873	4120888	*hslU*
CTGTAACCGTTTTCAC	-	4124535	4124550	*priA*
CGGCAAGCGCTTTCAC	-	4227089	4227104	*metH*
**GGGTAATCGCGTCCAC**	**-**	**4256787**	**4256802**	***dgkA***
**GTGCAAAAGATTGCAC**	**-**	**4281671**	**4281686**	***yjcE***
**GGGTAATCGGTTTTAC**	**-**	**4330520**	**4330535**	***proP***
GTGGTTACGCTTTCAC	+	4338250	4338265	*adiA*
GCGTAATCGCTTTTAC	+	4377990	4378005	*ampC*
**GAGAAAACGCTTCAAC**	**-**	**4378149**	**4378164**	***ampC***

Table S1 continued

**CTGGCATCGTTTACAC**	**-**	**4433627**	**4433642**	***qorB***
ATGAAAGCGATTACAA	+	4449817	4449832	*ytfQ*
**AAGTAAGCGTTTCCAC**	**-**	**4449964**	**4449979**	***ytfQ***
**TTGCCACCGCTTTCAC**	**-**	**4483949**	**4483964**	***holC***
GTGGATCCAGTTGCAC	+	4555115	4555130	*uxuR*

Note: The motifs in bold letters are defined as Gene Regulatory Sites (GRS).
