# Supplementary material for: Genome-Wide Transcriptional Regulation and Chromosome Structural Arrangement by GalR in E. coli
Source: Front Mol Biosci. 2016 Nov 16;3:74. doi: 10.3389/fmolb.2016.00074 (PMC5110547; doi:10.3389/fmolb.2016.00074)
Supplement: Supplementary file 2 [file Table2.PDF]

Table S2: Transcription Level of Genes in ΔgalR Strain Relative to Wild type GalR

Gene Number	Gene Name	Fold change ΔgalR/WT		Gene Number	Gene Name	Fold change ΔgalR/WT
b0759	*galE*	92.088 up		b0032	*carA*	5.201 down
b0758	*galT*	49.659 up		b1717	*rpmI*	5.140 down
b2943	*galP*	19.678 up		b2587	*kgtP*	5.118 down
b0757	*galK*	19.028 up		b3123	*rnpB*	5.077 down
b2518	*ndk*	7.217 down		b0721	*sdhC*	5.021 down
b3186	*rplU*	7.153 down		b1716	*rplT*	5.007 down
b3065	*rpsU*	6.470 down		b2185	*rplY*	4.951 down
b3636	*rpmG*	6.317 down		b2313	*cvpA*	4.876 down
b2000	*flu*	6.307 down		b3298	*rpsM*	4.862 down
b1677	*lpp*	6.011 down		b3977	*tyrU*	4.851 down
b0023	*rpsT*	5.996 down		b1237	*hns*	4.835 down
b3637	*rpmB*	5.969 down		b0623	*cspE*	4.785 down
b1089	*rpmF*	5.886 down		b0907	*serC*	4.743 down
b0911	*rpsA*	5.501 down		b1088	*yceD*	4.739 down
b1094	*acpP*	5.461 down		b3185	*rpmA*	4.676 down
b0743	*lysT*	5.454 down		b1415	*aldA*	4.672 down
b0756	*galM*	5.335 up		b2590	*gltW*	4.628 down
b0432	*cyoA*	5.322 down		b0722	*sdhD*	4.614 down
b4435	*isrC*	5.293 down		b2499	*purM*	4.599 down
b1892	*flhD*	5.256 down		b4202	*rpsR*	4.588 down
b0741	*pal*	5.226 down		b2913	*serA*	4.557 down

Table S2 Continued

Gene Number	Gene Name	Fold change ΔgalR/WT		Gene Number	Gene Name	Fold change ΔgalR/WT
b0742	*ybgF*	4.498 down		b3738	*atpB*	4.031 down
b4506	*ykgO*	4.463 down		b2153	*folE*	4.028 down
b0744	*valT*	4.418 down		b4414	*tff*	4.026 down
b1077	*flgF*	4.386 down		b3969	*gltT*	4.019 down
b1891	*flhC*	4.383 down		b0953	*rmf*	4.018 down
b3296	*rpsD*	4.349 down		b0746	*valZ*	4.003 down
b1078	*flgG*	4.324 down		b3737	*atpE*	3.986 down
b3908	*sodA*	4.271 down		b3985	*rplJ*	3.985 down
b4201	*priB*	4.254 down		b1778	*msrB*	3.977 down
b1076	*flgE*	4.207 down		b3986	*rplL*	3.971 down
b1761	*gdhA*	4.177 down		b0557	*borD*	3.970 down
b3978	*glyT*	4.167 down		b0429	*cyoD*	3.939 down
b1603	*pntA*	4.142 down		b3165	*rpsO*	3.923 down
b0745	*lysW*	4.141 down		b3736	*atpF*	3.885 down
b1452	*yncE*	4.141 down		b3310	*rplN*	3.875 down
b0296	*ykgM*	4.131 down		b2942	*metK*	3.861 down
b3297	*rpsK*	4.119 down		b4015	*aceA*	3.848 down
b0672	*leuW*	4.077 down		b4200	*rpsF*	3.842 down
b1093	*fabG*	4.063 down		b0436	*tig*	3.833 down
b0203	*alaV*	4.043 down		b3312	*rpmC*	3.830 down
b2609	*rpsP*	4.034 down		b3256	*accC*	3.826 down

Table S2 Continued

Gene Number	Gene Name	Fold change ΔgalR/WT		Gene Number	Gene Name	Fold change ΔgalR/WT
b1831	*proQ*	3.812 down		b4355	*tsr*	3.666 down
b1946	*fliN*	3.808 down		b3309	*rplX*	3.665 down
b2093	*gatB*	3.800 down		b3313	*rplP*	3.654 down
b4314	*fimA*	3.790 down		b0928	*aspC*	3.648 down
b2911	*ssrS*	3.789 down		b0723	*sdhA*	3.633 down
b3759	*rrfC*	3.786 down		b3300	*secY*	3.631 down
b1073	*flgB*	3.769 down		b2175	*spr*	3.610 down
b4147	*efp*	3.753 down		b3302	*rpmD*	3.609 down
b2402	*valX*	3.749 down		b0670	*glnU*	3.598 down
b0755	*gpmA*	3.745 down		b3958	*argC*	3.596 down
b0407	*yajC*	3.730 down		b0727	*sucB*	3.594 down
b3979	*thrT*	3.723 down		b0178	*hlpA*	3.589 down
b1882	*cheY*	3.722 down		b1925	*fliS*	3.586 down
b0440	*hupB*	3.708 down		b0754	*aroG*	3.580 down
b3295	*rpoA*	3.698 down		b0471	*ybaB*	3.575 down
b1718	*infC*	3.694 down		b1782	*mipA*	3.574 down
b3255	*accB*	3.686 down		b0747	*lysY*	3.570 down
b3433	*asd*	3.683 down		b3299	*rpmJ*	3.565 down
b0740	*tolB*	3.672 down		b3703	*rpmH*	3.551 down
b0525	*ppiB*	3.671 down		b4008	*gltV*	3.551 down
b4408	*csrB*	3.670 down		b3231	*rplM*	3.545 down

Table S2 Continued

Gene Number	Gene Name	Fold change ΔgalR/WT		Gene Number	Gene Name	Fold change ΔgalR/WT
b1092	*fabD*	3.543 down		b3294	*rplQ*	3.395 down
b1194	*ycgR*	3.531 down		b1881	*cheZ*	3.387 down
b2608	*rimM*	3.526 down		b2606	*rplS*	3.386 down
b3315	*rplV*	3.521 down		b2416	*ptsI*	3.384 down
b1945	*fliM*	3.518 down		b3735	*atpH*	3.379 down
b3303	*rpsE*	3.505 down		b2476	*purC*	3.359 down
b1857	*znuA*	3.504 down		b4014	*aceB*	3.358 down
b2607	*trmD*	3.494 down		b3314	*rpsC*	3.357 down
b3261	*fis*	3.454 down		b3734	*atpA*	3.357 down
b3342	*rpsL*	3.449 down		b1777	*yeaC*	3.355 down
b0169	*rpsB*	3.448 down		b3976	*thrU*	3.352 down
b0553	*nmpC*	3.444 down		b1075	*flgD*	3.351 down
b3311	*rpsQ*	3.444 down		b0814	*ompX*	3.347 down
b2239	*glpQ*	3.438 down		b3230	*rpsI*	3.341 down
b3260	*dusB*	3.428 down		b0880	*cspD*	3.327 down
b2551	*glyA*	3.425 down		b2007	*yeeX*	3.327 down
b3306	*rpsH*	3.423 down		b3316	*rpsS*	3.321 down
b1244	*oppB*	3.414 down		b1602	*pntB*	3.307 down
b1136	*icd*	3.409 down		b1888	*cheA*	3.307 down
b2094	*gatA*	3.403 down		b2621	*ssrA*	3.307 down
b2926	*pgk*	3.403 down		b3319	*rplD*	3.304 down
b1074	*flgC*	3.396 down		b1480	*sra*	3.301 down

Table S2 Continued

Gene Number	Gene Name	Fold change ΔgalR/WT		Gene Number	Gene Name	Fold change ΔgalR/WT
b3732	*atpD*	3.299 down		b0116	*lpd*	3.225 down
b3853	*alaT*	3.298 down		b1818	*manY*	3.225 down
b0431	*cyoB*	3.296 down		b3308	*rplE*	3.225 down
b0205	*rrfH*	3.291 down		b3321	*rpsJ*	3.222 down
b1885	*tap*	3.291 down		b2925	*fbaA*	3.216 down
b0605	*ahpC*	3.288 down		b1207	*prsA*	3.215 down
b1044	*ymdA*	3.283 down		b4177	*purA*	3.213 down
b3983	*rplK*	3.274 down		b4226	*ppa*	3.213 down
b2529	*iscU*	3.273 down		b0728	*sucC*	3.209 down
b1923	*fliC*	3.268 down		b3984	*rplA*	3.209 down
b0889	*lrp*	3.262 down		b3318	*rplW*	3.208 down
b0724	*sdhB*	3.261 down		b2288	*nuoA*	3.206 down
b2530	*iscS*	3.260 down		b2480	*bcp*	3.204 down
b0118	*acnB*	3.258 down		b0172	*frr*	3.197 down
b3307	*rpsN*	3.252 down		b0729	*sucD*	3.190 down
b3760	*aspT*	3.252 down		b3304	*rplR*	3.189 down
b2092	*gatC*	3.240 down		b3733	*atpG*	3.186 down
b0748	*lysZ*	3.238 down		b0726	*sucA*	3.171 down
b0957	*ompA*	3.237 down		b0430	*cyoC*	3.169 down
b1071	*flgM*	3.236 down		b1924	*fliD*	3.168 down
b1324	*tpx*	3.231 down		b3981	*secE*	3.162 down

Table S2 Continued

Gene Number	Gene Name	Fold change ΔgalR/WT		Gene Number	Gene Name	Fold change ΔgalR/WT
b1288	*fabI*	3.148 down		b2027	*cld*	3.059 down
b2403	*valY*	3.117 down		b1612	*fumA*	3.057 down
b1083	*flgL*	3.102 down		b0166	*dapD*	3.054 down
b1886	*tar*	3.096 down		b0004	*thrC*	3.044 down
b3301	*rplO*	3.095 down		b3426	*glpD*	3.034 down
b0415	*ribE*	3.088 down		b2310	*argT*	3.027 down
b0720	*gltA*	3.086 down		b0668	*glnW*	3.022 down
b3757	*gltU*	3.082 down		b2401	*valU*	3.015 down
b1334	*fnr*	3.080 down		b3739	*atpI*	3.007 down
b1920	*fliY*	3.080 down		b1973	*yodA*	3.005 down
b3774	*ilvC*	3.071 down		b3276	*alaU*	3.004 down
b0180	*fabZ*	3.060 down		b2531	*iscR*	3.001 down
b1079	*flgH*	3.060 down				
